# Arginine Signaling and Cancer Metabolism

**DOI:** 10.3390/cancers13143541

**Published:** 2021-07-15

**Authors:** Chia-Lin Chen, Sheng-Chieh Hsu, David K. Ann, Yun Yen, Hsing-Jien Kung

**Affiliations:** 1 Institute of Molecular and Genomic Medicine, National Health Research Institutes, Zhunan 350, Miaoli County, Taiwan; truip75@gmail.com; 2 Institute of Biotechnology, National Tsing-Hua University, Hsinchu 30035, Taiwan; b8921019@gmail.com; 3 Institute of Cellular and System Medicine, National Health Research Institutes, Zhunan 350, Miaoli County, Taiwan; 4 Department of Diabetes and Metabolic Diseases Research, Irell & Manella Graduate School of Biological Sciences, Beckman Research Institute, City of Hope, Duarte, CA 91010, USA; dann@coh.org; 5 Ph.D. Program for Cancer Biology and Drug Discovery, College of Medical Science and Technology, Taipei Medical University, Taipei 110, Taiwan; yyen@tmu.edu.tw; 6 Research Center of Cancer Translational Medicine, Taipei Medical University, Taipei 110, Taiwan; 7 Comprehensive Cancer Center, Department of Biochemistry and Molecular Medicine, University of California at Davis, Sacramento, CA 95817, USA

**Keywords:** arginine, cancer metabolism, epigenetics, arginine-deprivation therapy, ADI, arginase

## Abstract

**Simple Summary:**

In this review, we describe arginine’s role as a signaling metabolite, epigenetic regulator and mitochondrial modulator in cancer cells, and summarize recent progress in the application of arginine deprivation as a cancer therapy.

**Abstract:**

Arginine is an amino acid critically involved in multiple cellular processes including the syntheses of nitric oxide and polyamines, and is a direct activator of mTOR, a nutrient-sensing kinase strongly implicated in carcinogenesis. Yet, it is also considered as a non- or semi-essential amino acid, due to normal cells’ intrinsic ability to synthesize arginine from citrulline and aspartate via ASS1 (argininosuccinate synthase 1) and ASL (argininosuccinate lyase). As such, arginine can be used as a dietary supplement and its depletion as a therapeutic strategy. Strikingly, in over 70% of tumors, ASS1 transcription is suppressed, rendering the cells addicted to external arginine, forming the basis of arginine-deprivation therapy. In this review, we will discuss arginine as a signaling metabolite, arginine’s role in cancer metabolism, arginine as an epigenetic regulator, arginine as an immunomodulator, and arginine as a therapeutic target. We will also provide a comprehensive summary of ADI (arginine deiminase)-based arginine-deprivation preclinical studies and an update of clinical trials for ADI and arginase. The different cell killing mechanisms associated with various cancer types will also be described.

## 1. Introduction

An important step in tumor development is a metabolic adaptation to cope with the demand of rapid cell division as well as a hypoxia, and nutritionally deprived microenvironment [1]. Different tumors utilize different strategies to reprogram their metabolic pathways. In so doing, tumor cells expose specific vulnerabilities, which can be exploited therapeutically. For instance, tumor cells, not their normal counterparts, are “addicted” to certain external nutrients including amino acids and amino acid starvation therapy has gained significant momentum in recent years [2]. One of the most common metabolic defects of tumor cells is the impaired intrinsic ability to synthesize arginine [3]. Targeting exogenous arginine by arginine-metabolizing enzymes such as arginase, arginine decarboxylase and arginine deiminase (ADI) has received increasing attention as therapies to treat a variety of cancers [4]. There are a number of excellent reviews on this topic [5,6,7]. In this review, we will focus on recent progress in understanding arginine’s role in cancer metabolism as a signaling metabolite, an epigenetic regulator and an immunomodulator. As much of the knowledge was derived from characterizing arginine-deprived cancer cells, we will also update the current status of arginine-deprivation therapy.

## 2. Arginine and Cancer Metabolism

Arginine is involved in numerous biological functions including cell proliferation, cell signaling, muscle contraction, immunity, neurotransmission, vasodilation, synthesis of growth factors and other amino acids [8]. Three major resources of arginine include (1) arginine-enriched nutrition supplement from dietary intake, such as chicken, pork loin, pumpkin seeds, peanuts, soybeans and so on (approximately 5 g of arginine per day), (2) endogenous synthesis from citrulline (15% of the total arginine production) and (3) protein catabolism (approximately 80% of the circulating arginine) [9]. Arginine is a direct resource for nitric oxide (NO), ornithine and agmatine through three enzyme reactions. Arginine is metabolized into NO and citrulline by nitric oxide synthase (NOS), into ornithine and urea by arginase, and into agmatine by arginine decarboxylase (ADC). NO serves multiple physiological functions.

**Figure 1:**
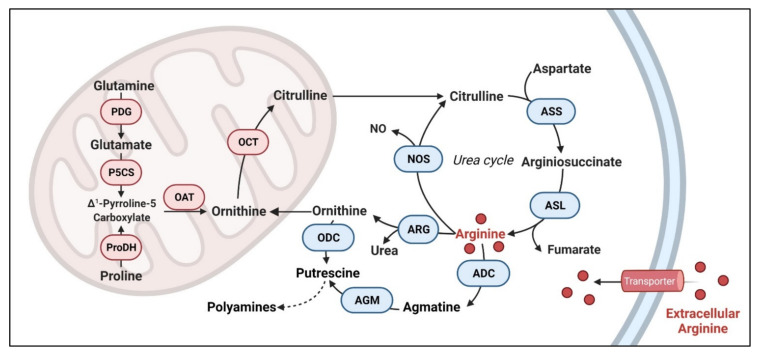
Arginine-related metabolic pathways. In normal cells, arginine can be directly transported into the cell or synthesized from citrulline and aspartate in the urea cycle. The resources of citrulline include glutamine, glutamate and proline. Arginine can be converted into nitric oxide (NO), urea and agmatine. Agmatine and ornithine are important resource for putrescine, which is a key precursor for polyamines. ASS: argininosuccinate synthetase, ASL: argininosuccinate lyase, ADC: arginine decarboxylase, AGM: agmatinase, ODC: ornithine decarboxylase, ARG: arginase, NOS: nitric oxide synthase, OCT: ornithine carbamoyl transferase, OAT: ornithine aminotransferase, PDG: phosphate-dependent glutaminase, P5CS: pyrroline-5-carboxylate synthase, ProDH: proline dehydrogenase.

Both ornithine and agmatine are the main resources for putrescine, which is a crucial precursor for polyamines [10] (Figure 1). These metabolites play key functions in cell physiology and human health so does arginine. Yet, arginine is also considered a “semi-essential” or “conditional essential” amino acid. This is due to normal cells’ intrinsic ability to synthesize this molecule from citrulline and aspartate via argininosuccinate synthase 1 (ASS1) and argininosuccinate lyase (ASL) in the urea cycle [11]. Citrulline can be synthesized from glutamate [12], proline [13] and glutamine [14]. As such, normal cells do not completely depend on external arginine. Yet, many cancer cells are “addicted” to external arginine.

Much of our current understanding of the arginine effects on cancer metabolism is derived from arginine-deprivation studies of ASS1-low cancer cells. The responses depend on the cell types studied but generally can be put into two categories.

For breast and prostate cancer cells, arginine starvation causes global transcriptional suppression of metabolic genes including those involved in oxidative phosphorylation (OXPHOS) and mitochondrial functions, glycolysis, purine and pyrimidine synthesis, DNA repair genes [15,16,17,18], due to epigenetic remodeling [19,20]. Removal of arginine causes fragmentation of mitochondria and impairment of mitochondrial functions as measured by OCR (oxygen consumption rate) and membrane potential [15,17,20,21]. The suppressed transcription of genes involved in mitochondria functions is reflected by the metabolomics studies which show the general depletion of TCA cycle metabolites such αKG, malate, fumarate and succinate [17,20]. It was suggested that at least in breast cancer cells, such depletions are in part due to the ER-stress mediated activation of ASNS (asparagine synthetase), which depletes aspartate and diminishes aspartate-malate shuttle, negatively impacting TCA [17]. The perturbation of OXPHOS reaction due to metabolite depletion, perpetuated by the transcriptional suppression of nuclear-encoded OXPHOS genes, generates copious amount of mtROS which leads to DNA damage and eventual cell death [15,16,17,20]. Indeed, in these cells, functional knockout of mitochondria prevented DNA damage and cell death [17]. Additional evidence for impairment of mitochondria by arginine deprivation includes the morphological changes from hyperfusion at early stage to fragmentation at late state [17,20]. This transition is caused by the reduced expression of Mfn2 (mitofusin2) mediated by arginine deprivation-induced p38 activation and KAP-1 phosphorylation [21].

By contrast, arginine deprivation of ASS1-low melanoma and sarcoma cells leads to downmodulation of glycolysis pathway with increased glutamine anaplerosis and serine synthesis to sustain the TCA cycle [22,23]. The authors suggest the inhibition of the Warburg effect is one reason how cancer cells adapt to the stress environment and eventually become resistant to the treatment. A characteristic of these cells is the activation of c-myc upon arginine deprivation [24,25], which is not necessarily the case in prostate and breast cancers described above and may explain the differences in their responses to arginine deprivation. c-myc is known to upregulate glutaminase and render cells addicted to glutamine [26]. Accordingly, these types of tumors are synthetic lethal with glutaminase inhibitor [22].

In either of the above cases, mitochondria are targeted by arginine deprivation and arginine represents a major regulator of mitochondrial activities in cancer metabolism.

## 3. Arginine and Signal Transduction

### 3.1. Arginine Mediated Signals

**Figure 2:**
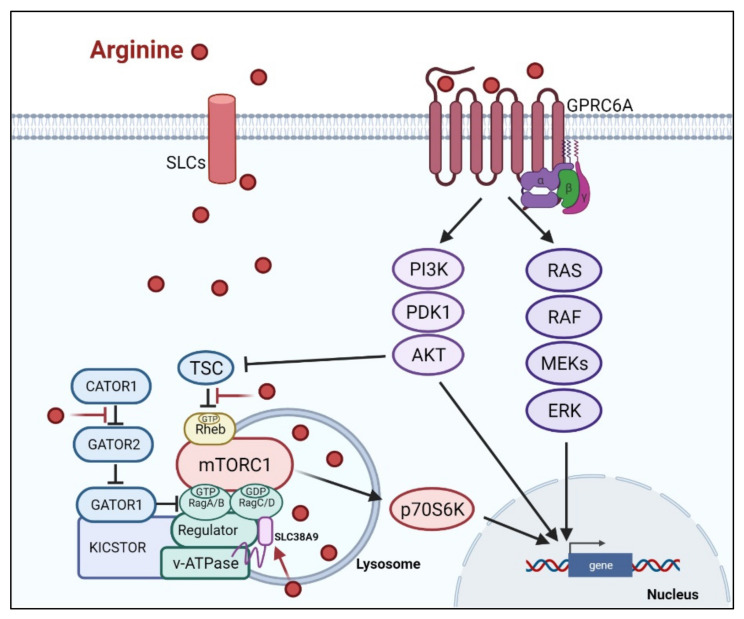
Arginine-related signaling pathways. Arginine can be transported by solute carriers (SLCs), including lysosomal SLC38A9, to activate the down-stream mTORC1 signaling pathway. In addition, arginine also can bind G-protein coupled receptor, GPRCA6, to activate down-stream RAS/ERK or PI3K pathway to reprogram the general.

There are at least two ways arginine can transmit signals to the cells. The first is through transporters, solute carriers (SLCs) (Figure 2). As a cationic amino acid, arginine is mainly imported by two types of SLCs, the cationic amino acid transporters and the system y^+^L amino acid transporters [27,28]. It is noteworthy that arginine activates its downstream mTOR signal via lysosomal SLC38A9 [29]. Arginine is the most consumed amino acid in the inner necrotic core of tumor mass, indicating its high demand for the survival of tumor cells [30]. Accordingly, tumor cells frequently overexpress specific types of SLCs such as SLC6A14, SLC7A3, SLC7A9, etc. to meet their high arginine demand (Table 1). It should be noted that T cells up-regulate distinct types of SLCs to increase arginine uptake for T cell activation and anti-tumor functions [31,32] (Table 1). Thus, targeting the tumor specifically with SCLs, and avoiding those expressed in T-cell and macrophage (e.g., SLC7A1, and A2) could be a potential strategy for cancer therapy.

Subsequent to its transport, arginine is able to activate several signal pathways. Chief among them is mTOR kinase. Arginine is one of only three amino acids that can directly activate mTOR pathway, a major cellular sensor of nutritional state [47]. The other two are glutamine and leucine. As such, arginine has profound impacts on protein synthesis, lipid synthesis and nucleotide synthesis, three anabolic pathways mediated by mTOR [48]. Indeed, nutrients [49,50]. are as important as growth factors in the activation of mTOR. Upon growth factor stimulation, mTOR can be activated through either PI3K (phosphatylinositol 3-kinase) pathway or MAPK pathway, via the inactivation of TSC (tuberous sclerosis complex), an mTOR negative regulator [51]. Inhibition of TSC converts the Rheb (RAS homolog enriched in brain) into active form, resulting in the activation of mTORC1 (mTOR complex 1). There are at least three ways, arginine can activate mTOR. (1) arginine disrupts the interaction between TSC and mTORC1, thereby activating mTOR [52]. (2) in the lysosome, arginine interacts with SLC38A9 and v-ATPase, upstream regulators of mTORC1, leading to the activation of Rag GTPase that is required for recruitment of mTORC1 complex to the lysosomal surface [53,54]. (3) in the cytosol, arginine interacts with CASTOR1(cytosolic arginine sensor for mTORC1 subunit 1) to disrupt the CASTOR complex, which is a negative regulator of Rag A [55], allowing Rag A to bind mTORC1 component RAPTOR (Regulatory-associated protein of mTOR) and redistributes mTORC1 to the lysosome. This may explain why arginine is such a potent activator of mTOR, and arginine deprivation leads to immediate inactivation of mTOR.

The second means arginine can transmit signal is through binding to L-amino acid receptor, G-protein coupled receptor GPRCA6 [56]. In human fibroblast, GPRCA6 was shown to activate PKA (activate protein kinase A), RAS/ERK (extracellular regulated protein kinases) and PI3K/Akt/mTOR pathways [57,58] and in human prostate cancer cell line, arginine/GPRCA6 pathway stimulates ERK pathway to mediate its growth and progression [59].

There are additional ways that arginine may modulate cellular signal pathways. As mentioned, arginine is a precursor to NO, by the action of NO synthase (NOS) [60]. The duality of NO functions as a signaling messenger in cancer has been noted [61]. NO was regarded as an anti-tumor reagent due to its antioxidant capacity and radical scavenging property [62]. Emerging evidence, however, shows a diverse role of NO in tumorigenesis, including angiogenesis, metastasis, anti-apoptosis, anti-host immune response [63]. The increased level of NO and NOS expression has been observed in cancer patients, which is highly correlated with VEGF expression, angiogenesis [64] and metastasis [65]. Induction of NO and NOS leads to the inactivation of tumor suppressors, such as p53 [66] and pRb [67]. It has also been shown that induction of NOS and cyclooxygenase (COX-2) increased the level of NO and prostaglandins, which leads to angiogenesis in cancers [68,69,70]. Another way NO exerts its effect is through S-nitrosylation of key molecules involved in cancer induction such as EGFR and TSC2 which impacts mTOR pathway [71,72].

In addition to cell growth, NO affects immune cells in tumor microenvironment and again, in a dual mode. The dual roles of NO in carcinogenesis notwithstanding, arginine is generally considered as a tumor-promoting metabolite, very much needed for tumor growth [73].

### 3.2. Arginine Deprivation Induced Signals

**Figure 3:**
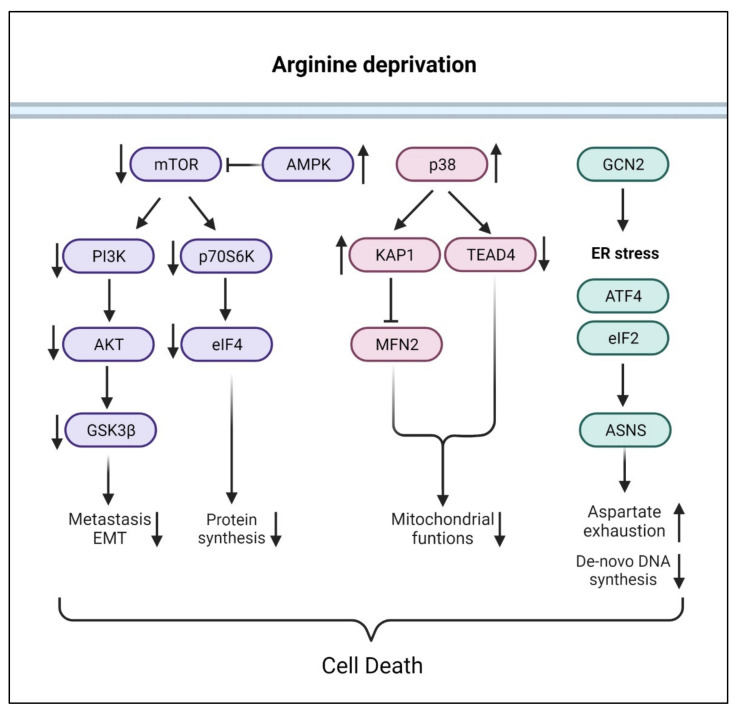
Arginine deprivation signaling pathways. In contrast to arginine stimulation, arginine deprivation suppresses mTOR pathway via activation of AMPK. Additionally, arginine deprivation induces p38-signaling pathway, which impairs mitochondria functions. Arginine deprivation also can induce ER stress via GCN2, resulting in aspartate exhaustion and decreased DNA synthesis. In general, arginine deprivation causes cancer cell death. EMT: epithelial-mesenchymal transition.

Consistent with arginine being an activator of mTOR and PI3K pathways, numerous studies showed that arginine-deprivation suppresses mTOR and p70S6K activation with consequent inactivation of PI3K/Akt pathway [74,75] (Figure 3). In addition, arginine-deprivation activates AMPK (5’ adenosine monophosphate-activated protein kinase), due to the reduced mitochondrial OXPHOS activities and ATP production, which further suppresses mTOR activities through inactivating phosphorylation [75]. The consequences of severe mTOR suppression include aggressive autophagy and diminished synthesis of proteins, lipids and nucleotides [75,76].

Arginine deprivation also activates stress-response kinase p38, which impacts mitochondria functions, by (1) activating KAP1 (KRAB-associated protein 1) and mitochondrial fission [21] and (2) translocates nuclear TEAD4 (TEA Domain Transcription Factor 4) to cytosol, resulting in the reduced nuclear OXPHOS gene expression and mitochondrial functions [19].

Arginine deprivation in the form of nutritional stress-mediated GCN2 (general control nonderepressible 2) activation and ER stress pathway, leading to ATF4 (activating transcription factor 4) and ASNS activation and increased consumption of aspartate. Aspartate exhaustion is one reason arginine-deprivation causes the death of ASS1-low cancer cells [17].

## 4. Arginine and Epigenetic Regulation

Recent studies showed that arginine can act as an effective epigenetic modulator [19]. In cancer cells, arginine is a strong inducer of histone acetylation, globally enhancing the expression of metabolic, mitochondrial and DNA repair genes. Histone acetylation involves the transfer of acetyl group from acetyl-CoA to histone mediated by HATs (histone acetyltransferases) and KATs (lysine acetyltransferases), which is counteracted by deacetylation enzymes such as HDACs (histone deacetylases) and SIRTs (sirtuins). Several enzymes including ACLY (ATP citrate synthase), ACSS1 (Acyl-coA synthetase short-chain family member 1) and ACSS2 (Acyl-coA synthetase short-chain family member 2) contribute to the synthesis of acetyl-CoA. In arginine stimulated cells, the acetyl-CoA level significantly increases so do the expression levels of ACLY, ACSS2 and the majority of HATs and KATs. By contrast, the expressions of several of the HDACs and SIRTs are decreased. These results together could account for the increased global histone acetylation observed. Since mTOR is known to activate ACLY and ACSS [77,78], arginine stimulation of histone acetylation is in part attributed to the activation of mTOR. The global increase of histone acetylation however is not random but has region specificity, which is dictated by several transcription factors including TEAD4, STAT3 (signal transducer and activator of transcription 3), WT1 (Wilms’ tumor 1) and TFAM (mitochondrial transcription factor A).

**Figure 4:**
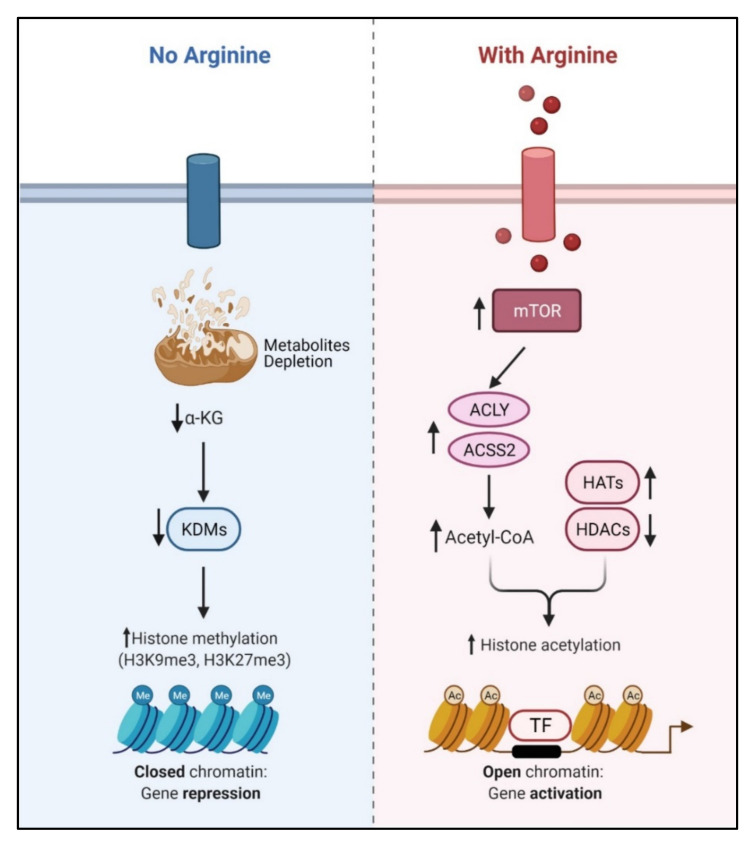
Arginine acts as an epigenetic regulator. In the presence of arginine, mTOR induces the ACLY and ACSS2 to increase the level of acetyl-CoA, which is the main resource of histone acetyl-transferases (HATs). Increased histone acetylation induces the chromatin-remodeling and gene activation. Conversely, arginine deprivation causes metabolites depletion, including alpha-ketoglutarate (α-KG), which down-regulates lysine-demethylases (KDMs) and induces globe repressive histone methylations.

Conversely, arginine deprivation leads to depletion of α-KG, which has profound effects on epigenetic regulation. As described above, arginine deprivation immediately affects mitochondrial functions [15,17,20,21] with consequent depletion of mitochondrial metabolites including α-KG. Alpha-KG is a cofactor of jumonji domain C containing histone demethylases (KDMs). As such, histone methylation generally increases during arginine deprivation. Most prominent are H3K9me3 and H3K27me3, two repressive marks contributing to gene silencing. These marks decorate genes involved in mitochondrial functions including OXPHOS, purine and pyrimidine synthesis, DNA repair, etc. (Figure 4). The consequence of such epigenetic repression is mitochondrial dysfunction, generation of reactive oxygen species (ROS), DNA damage and slow DNA repair, features which figure prominently in arginine-deprived tumor cells [15,17,20].

## 5. Arginine and Genome Integrity

Sufficient arginine is required for maintaining nucleotide pool and DNA repair capacity. Although arginine is not directly involved in the synthesis of nucleotide, arginine can be converted to glutamine, proline and serine, precursors of pyrimidine and arginine abundance affects genome integrity. As described above, arginine augments the transcription of genes involved in purine and pyrimidine synthesis. In addition, by virtue of activating mTOR/S6K pathway, arginine promotes the phosphorylation and oligomerization of CAD complex (carbamoyl-phosphate synthetase 2, aspartate transcarbamylase, and dihydroorotase) to enhance pyrimidine synthesis [79,80]. On the other hand, both arginine and pyrimidine syntheses require aspartate and they “compete” for this metabolite. Tumor cells often augment pyrimidine synthesis by suppressing arginine synthesis via epigenetic silencing of ASS1 [81,82], the basis of arginine-deprivation therapy. As described above, arginine deprivation also activates ATF4/ASAN which converts aspartate to asparagine causing depletion of nucleotide pool. In this scenario, the cell death caused by arginine-deprivation can be partially rescued by the addition of aspartate or nucleotide precursors [17].

In addition to arginine’s ability to epigenetically regulate the transcription of DNA repair genes, arginine affects DNA repair through the synthesis of polyamines. Polyamines interact with negatively charged DNA and plays a key role in maintaining the genome stability [83]. Polyamine depletion impairs DNA repair [84] and sensitizes cancer cells to genotoxic reagents [85,86]. Consistently, arginine deprivation which significantly reduces the polyamine levels [87] synergizes with polyamine inhibitors in the killing of cancer cells [87].

As arginine deficiency both depletes nucleotide pool and slows down DNA repair in tumor cells, it is no surprise that arginine starved tumor cells exhibit extensive DNA damages [16,17,20]. In ASS1-low pancreatic ductal adenocarcinoma, arginine deprivation exacerbates the HDAC inhibition-induced downregulation of C-terminal-binding protein-interacting protein (CtIP), a key protein for homologous recombination, leading to DNA damage and cell death [82]. In prostate and pancreatic cancer cells, arginine-starvation induced caspase-independent autophagic cell death with the appearance of nuclear DNA leakage and chromatin-autophagy (chromatophagy) [16]. This is caused by mitochondrial dysfunction and ROS production in the presence of excessive autophagy. Depletion of mitochondria or removal of ROS by NAC attenuates the DNA leakage phenotype and cell death [17]. In a study of ASS1-low melanomas, regardless of the BRAF status, arginine deprivation down-modulates FANCD2 and p-ATM, which are important initiators for DNA double strand break repair [88]. Although in this cell type, arginine deprivation alone does not induce DNA damage, combined treatment with cisplatin increases DNA double breaks, possibly due to persistent downregulation of DNA repair machinery caused by arginine deprivation. Taken together, arginine affects nucleotide synthesis/DNA repair in a complex way. The nucleotide insufficiency and down-modulated DNA repair machinery may underlie the arginine deprivation-induced DNA damage and its deficiency impairs this process and causes death of tumor cells.

## 6. Arginine and Immunomodulation

Arginine is a crucial immune-modulating amino acid for both innate and adaptive immunity [89,90]. It is involved in the activation of T-cell via the upregulation of T-cell receptor [91] and accelerating cell cycle progression [32]. Depletion of arginine has been used by tumor cells to generate an immunosuppressive micro-environment. Cancer cells release factors (G-CSF, GM-CSF, CCl2, etc.) to convert myeloid cells into immunosuppressive phenotypes (e.g., MDSC, myeloid-derived suppressive cells, or M2 macrophages) [92]. These cells are characterized by the expression and release of arginase [93]. Arginase produced by macrophages and MDSCs depletes extracellular arginine and hence suppression of T cell proliferation [94,95]. Supplement of arginine rewires T cell metabolism from glycolysis to OXPHOS and promotes its survival and anti-tumor ability [96]. Arginase inhibitors thus have been developed and under clinical trials as cancer therapy to counteract such immune suppression [97]. This presents an interesting duality of arginase as cancer therapy. On the one hand, arginase itself can effectively starve ASS1-low tumor cells to death. On the other, arginase inhibitor could help boost the immune defense system to suppress the growth of cancer cells. Arginine also affects innate immunity primarily via the synthesis of NO. Besides acting as a neurotransmitter and vasodilator, at high concentration, NO’s radical property enables spontaneous reactions with oxygen to form reactive nitrogen oxide species (RNOS) [98]. These RNOS reacts with DNA, protein and lipid causing DNA damage, protein dysfunction, and lipid peroxidation, which contributes to the antimicrobial and tumoricidal activities [99]. NO’s synthesis is limited by arginine availability. NO is synthesized by inducible nitric oxide synthase (iNOS) in M1 macrophages, which converts arginine into citrulline and NO (Figure 1). NO induces metabolic rewiring in M1 macrophages [100], and inhibition of iNOS induces M1 polarization into M2 type [101]. Indeed, the two arginine-metabolizing enzymes, iNOS from M1 macrophage and arginase from M2 macrophage compete for arginine to modulate innate immune responses [90]. In addition to cancer cells, several infectious bacteria and parasites also utilize the same strategy to create immunosuppressive niche by enhancing arginase expression in M2 macrophages, such as mycobacteria, Helicobacter pylori and Leishmania major [102,103,104].

Although the correlation between arginine removal by arginase and T cell suppression has been well established, how systemic removal of arginine affects the tumor microenvironment and tumor growth remains poorly understood. In a study of the effect of autophagy on tumor growth, it was found that autophagy-negative (Atg-/-) mouse released abundant arginase in the serum with consequent systemic depletion of arginine [73]. In these mice, ASS1-low syngeneic murine melanoma cells failed to grow with the infiltration of CD8 positive cells, indicating the T cell immune response is not severely affected by systemic depletion of arginine.

In another study, when human peripheral blood mononuclear cells (PBMCs) are stimulated by anti-CD3/CD28 antibodies, co-treatment with ADI-PEG20 does not block T cell activation; instead, arginine deprivation sustains the CD69+ T cells up to 72 h [105]. In the meantime, the induction of CTLA4 and PD-1 in activated T cells is blunted by arginine deprivation, and arginine deprivation also prevents Treg cells differentiation. Although arginine deprivation decreases cell proliferation of activated T cell as shown by previous studies, T cell infiltration is not compromised in syngeneic models [73,105].

Furthermore, using murine MC38 colon cell model, it was shown that ADI-PEG20 is able to induce apoptotic and immunogenic cell death with the phenotypes of cell exposure of calreticulin, the release of HMGA1 and ATP, which is enhanced by N-acetylcystein [106]. These “eat me” signals resulted in increased phagocytosis by bone marrow-derived dendritic cells. Another intriguing study shows that upon ADI-PEG20 treatment, the surrounding human T cells, which express ASS1, are still able to proliferate by taking up citrulline from the byproduct of ADI reaction and generate arginine intracellularly [107]. These studies, taken together, suggest arginine deprivation, despite its ability to suppress T-cells, are able to elicit immune responses.

More and more studies reveal that innate immunity is also crucial for anti-tumor responses [108], such as NK cell-mediated immunosurveillance [109]. In this regard, it is interesting that a recent study showed that arginine-deprivation activated innate immune response and turned “cold” tumors “hot” [20]. This is caused by cGAS-STING activation triggered by nuclear DNA leakage caused by arginine-starvation induced chromatin-autophagy. Preclinical models further showed the enhanced infiltration of immune cells in dietary arginine restricted animal model and this process is dependent on the activity of cGAS. Thus, arginine’s role in immunomodulation is complex and under certain circumstances, it may enhance the anti-tumor immunity.

Increasing evidence shows that arginine deprivation induces autonomous cancer cell death and enhances immune response. Dietary arginine-restriction offers a promising option for prevention and intervention.

Hitherto, we narrated the significance of arginine from the perspective of its role in tumorigenesis which reveals the potential of arginine deprivation as cancer therapy. In the ensuing sections, we will focus on arginine-deprivation therapy. We will first describe how arginine deprivation induces cancer cell death via different mechanisms and comprehensively summarize the current applications of arginine deprivation therapy on various cancers and discuss the associated challenges.

## 7. Arginine Deprivation and Cell Killing

**Figure 5:**
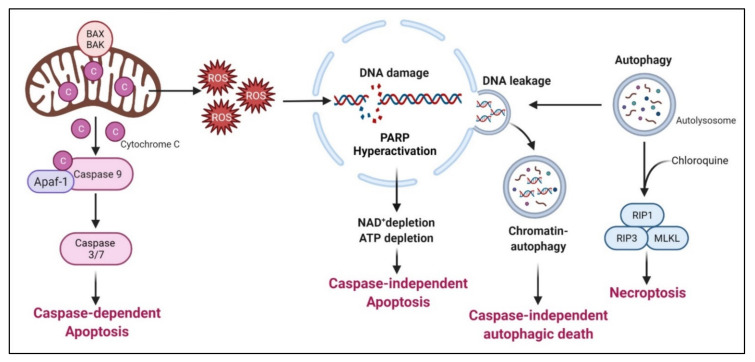
Arginine deprivation-induced types of cell death. The general cell-killing mechanisms by arginine deprivation include caspase-dependent apoptosis, caspases-independent apoptosis, caspase-independent autophagic cell death and necorptosis.

Arginine deprivation suppresses the growth and induces cell death of ASS1-low cancer cells. The general mechanisms associated with cell killing have been studied in a number of systems (Figure 5).

### 7.1. Caspase-Dependent Apoptosis

This is the major mechanism associated with arginine-deprivation induced cell death, which operates in many cancer types including pleural mesothelioma cells [110], lymphoma cells [111], pancreatic cancer cells [82,112], ovarian cancers [113], sarcoma cells [114], T-lymphoblastic leukemia cells [115], liver cancer cells [116] and melanoma [117].

### 7.2. Caspase-Independent Apoptosis

Syed N et al. and Kelly MP reported that in some glioma cells and small cell lung carcinoma respectively, arginine deprivation-induced apoptosis, but it is caspase-independent [118,119]. The detailed mechanism remains to be elucidated.

### 7.3. Caspase-Independent Autophagic Death

Arginine deprivation inhibits mTOR, which is a negative regulator of autophagy. Accordingly, arginine deprivation is often accompanied by aggressive autophagy. Autophagy is a major means to regenerate arginine, which protects cells from nutrient stress. However, prolonged arginine deprivation leads to excessive and aberrant autophagy. This, coupled with ROS-induced DNA damage, leads to chromatin-autophagy or chromatophagy, where autolysosome fused with nuclear membrane and “extracts” broken chromatin out of nucleus [16], eventually leading to caspase-independent cell death. This was observed in prostate cancer cells [16], breast cancer cells [15]; hepatocellular carcinoma cells [120] and pancreatic cells [121].

In general, arginine deprivation initially induces autophagy to protect cells from starvation and at the same time, generates ROS (due to mitochondria impairment) and DNA damages which trigger apoptosis. During this early phase, an autophagy inhibitor such as chloroquine would increase cell death and enhances the drug efficacies [121]. For some cancer cells, however, autophagy persists and captures damaged broken DNA, leading to nuclear DNA leakage and cell death [20].

### 7.4. Necroptosis

As described above, arginine-deprivation induces autophagy which initially exerts a protective role and co-treatment with the autophagy inhibitor, chloroquine, and can facilitate the cell death [75,111,114,118]. In one study [114], it was shown such a treatment activates RIP kinase cascade, leading to necroptosis. Genetical knock-down of RIP1 or RIP3 or pharmaceutical treatment with necroptosis inhibitor, necrostatin, can protect against the co-treatment mediated cell death [114].

## 8. Arginine Deprivation and Cancer Therapy

The suppressed expression of ASS1, mainly due to promoter methylation [111], is making it the most prevalent metabolic deficiency of cancer and rendering cancer cells “addicted” to external arginine [122]. Given the importance of arginine in cellular processes and being the most consumed amino acids in the inner mass of tumors, it is counter-intuitive that ASS1 is epigenetically suppressed in cancer cells. As described above, one possible explanation is that ASS1 diverts aspartate to arginine synthesis from pyrimidine/purine synthesis which is much needed for tumor cells [81]. In addition, recent reports that ASS1 has other tumor suppressor functions such as inhibiting AKT activity [15,16,123]. Several studies showed that overexpression of ASS1 in tumor cells suppresses cell growth and ASS1 behaves as a tumor suppressor [17,124]. At any rate, ASS1 deficiency is a selective trait of tumor cells, making arginine deprivation a highly promising therapeutic strategy. Currently, there are two arginine deprivation therapies under clinical trials: ADI-PEG20 (pegylated arginine deiminase) and PEG-BCT-100 (pegylated recombinant human arginase1). A comparison of these two reagents is shown in Table 2.

They both are effective in depleting serum arginine, yet they are not completely innocuous. As discussed above, ADI-PEG20 generates citrulline and ammonia, and citrulline is able to affect the surrounding immune cells to generate arginine. PEG-BCT-100 produces ornithine and urea, and ornithine can fuel tumor cells to generate polyamines. The concentrations of ammonia and urea also need to be monitored during treatment. Nevertheless, these reagents have been shown to be effective and safe. The promising prospect of using arginine deprivation to starve cancer cells to death has in recent years, fueled the preclinical studies, in vitro (cell lines) and in vivo (xenografts) on a variety of ASS1-low tumors.

### 8.1. Preclinical Studies

Table 3 is an updated summary of publications on the application of ADI to different cancer cell lines. While the detailed cell killing mechanisms may vary among different cell types (see next section), ADI effectively inhibits the growth and survival of ASS1-low tumor cells, and in those cases tested, the susceptibility to ADI is inversely correlated with the level of ASS1. Resistance to ADI is related to recovery of ASS1 expression. These studies provide strong evidence that ASS1 expression level is a predictor for arginine-deprivation therapy. The table also listed combination treatments of chemotherapeutic agents with ADI on different cell lines. ADI synergizes with cisplatin, oxaliplatin, docetaxel, gemcitabine, TMZ to enhance the killing effects and in some cases, help overcome resistance to these drugs.

### 8.2. Clinical Trials

Indeed, among the AA (amino acid) deprivation therapies, arginine deprivation is the most advanced (Table 4). The first-generation arginine-deprivation therapy was based on arginase which suffers from the low enzymatic activity and sub-optimal pH [7,153]. The second-generation arginine-deprivation therapy, include human-recombinant arginase I (PEG-BCT-100) and pegylated-ADI (ADI-PEG20) showed significant promises [126,154,155]. Considering the adverse effects of arginase I on immunity described above, the arginase inhibitor (CB-1158) is being evaluated in vivo [97] and clinical trials (Table 4). We will update the clinical trials of both ADI-PEG20 and PEG-BCT-100 in Table 4. There are more than 20 clinical trials of ADI-PEG20 on more than 12 types of cancer, which have been completed or are ongoing. In most cases, phase I/II were completed with an excellent safety profile (Table 4). Co-targeting with other anti-cancer therapy is being developed. Trials on hepatocellular carcinoma and mesotheliomas are the most advanced and have reached phase III. The phase III mesothelioma trial involving pemetrexed, cisplatin and ADI-PEG20 is ongoing and actively recruiting. The phase III hepatocellular carcinoma trial with ADI-PEG20 as monotherapy did not reach the intended goal [156]. One of possibilities is the treatment induces anti-ADI-PEG20 antibody, which neutralized the ADI-PEG20 [157]. Another possibility is due to the re-expression of ASS1 with prior treatment, such as sorafenib, which may reduce the efficacy of ADI-PEG20 [156]. The possible mechanism of drug resistance is discussed in the following section. Arginase clinical trials are mostly in phase I and II. In Table 4, we also included clinical trials for arginase inhibitors, which are intended to enhance the T-cell functions and anti-tumor activities.

## 9. Arginine Deprivation and Therapy Resistance

**Figure 6:**
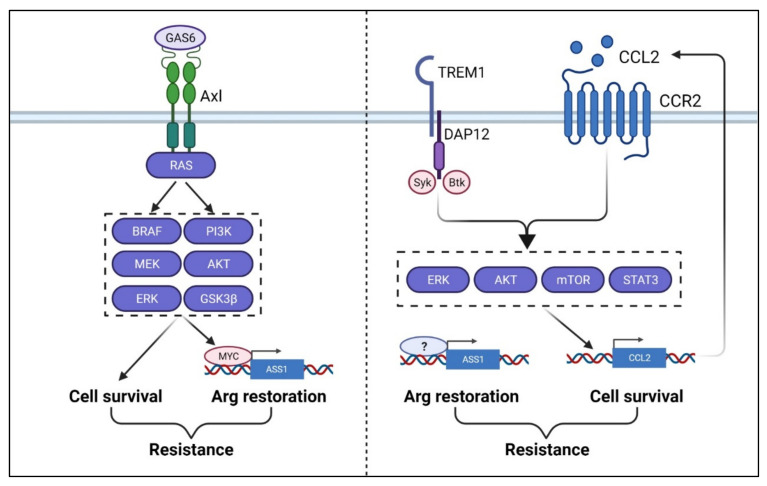
Therapy-resistance machinery. Upon arginine-depletion, TAM receptor, Axl, is activated by the growth arrest-specific protein 6 (Gas6), resulting in the activation of downstream signaling cascades. The activation of signaling pathways leads to MYC-induced ASS1 restoration and cell survival. In addition, the TREM1 induces CCL2 expression via ERK/AKT/mTOR/ STAT3 pathway, which leads to cell survival in ASS1-restoration cells, which also leads to the therapy-resistance.

One of the challenges in ADI-based therapy is the development of intrinsic resistance [170]. A common mechanism is the restoration or upregulation of ASS1 expression to provide much-needed arginine in starved cancer cells (Figure 6). For tumors (e.g., lymphomas, bladder, mesothelioma, prostate cancer) where ASS1 was silenced by methylation, demethylation of ASS1 promoter is the solution [87,111], although what triggers demethylation is not clear. For tumors of which the ASS1 promoter is not methylated (e.g., melanoma and sarcoma), the activation of c-Myc oncogene which drives the expression of ASS1 appears to be the underlying cause [22,23]. In the latter case, Gas6/Axl activation by arginine deprivation-induced ROS triggers PI3K/Akt/GSK3B activation, leading to the upregulation of c-Myc oncogene [152]. The studies by the lab of MT Kuo and his collaborators further demonstrated how c-Myc could replace negative regulator HIF-1a, which competitively occupied the same E-box of the ASS1 promoter to restore ASS1 expression [133]. On the other hand, c-Myc upregulation is not universally seen in all ADI-resistant cancer cells (e.g., prostate and breast cancer) and c-Myc overexpression does not always confer ADI resistance [18]. Interestingly, in lung cancer, Myc overexpression actually confers sensitivity, rather than resistance, to ADI treatment [139]. These studies suggest that while restoration of ASS1 expression seems to be necessary to provide the much-needed arginine during therapy, the contributing mechanisms vary and there are other context-dependent cellular factors associated with resistance. As mentioned above, ASS1 has been shown to repress Akt activities [123] and behaves as a tumor suppressor [15,124]. Other pathways are needed to offset its tumor suppressive properties. In melanoma, ADI-resistant cells, enhanced AXL and EPH2 tyrosine kinase activities and the activation of down-stream PI3K/AKT signaling are observed [152]. In breast and prostate cancer ADI-resistant cells, Chu et al., [18] using unbiased CRISPR/cas9 loss of function screening uncovered aberrant activation of TREM1/CCL2 is necessary to confer full resistance. TREM1, a receptor normally expressed in myeloid cells, activates tyrosine kinase Syk which turns on PI3K/Akt and STAT3/NFKB, leading to the activation of CCL2. CCL2, a chemokine known to confer drug resistance to a variety of cancer cells, further enhances the activation PI3K/Akt and STAT3/NFkB pathways [18]. As direct targeting ASS1 may affect the metabolism of normal cells, these resistant factors and their associated pathways offer additional opportunities as targets to overcome ADI resistance.

Khadeir et al. [170] further considered the factors which may compromise therapy efficacies in vivo, and suggests that in addition to ASS1 upregulation, protective autophagy which regenerates arginine, development of antibodies against pegylated ADI and the uptake of arginine released from stromal cells. Measures to counteract these resistant factors are being actively pursued to improve arginine-deprivation therapy.

## 10. Conclusions

As summarized in this article, arginine, in addition to being a building block of protein, can be a signaling metabolite, a transcriptome reprogrammer and a therapeutic target. Recent insights that arginine is a mitochondria and genome protector are exciting, with clinical implications. As a semi-essential amino acid, arginine deprivation based on biologicals which metabolize arginine has been a staple of starvation therapies for years. While the safety profiles for both arginine depletion remedies are generally excellent, as a monotherapy agent, it has not reached the intended potency. Combinations with other targeted or chemotherapies as well as immune checkpoint inhibitors are being actively pursued. Since the cell-killing mechanisms associated with starvation therapies (e.g., autophagic death, caspase-independent apoptosis, necroptosis, etc.) could be quite different from those associated with genotoxic agents (e.g., caspase-dependent apoptosis, mitotic catastrophe), arginine-deprivation should synergize well with other cancer therapies and potentially overcome the resistance of the latter. In addition, an arginine restriction diet also deserves serious attention as a complementary tool for cancer therapy. Unlike using metabolizing enzymes as depletion agents, dietary restriction does not have the complication of metabolic products. Dietary restriction has been widely applied in various metabolic diseases such as obesity, diabetes, rheumatoid arthritis, hypercholesterolemia, etc., and it has gained significant momentum for cancer therapies. Recent work showed that arginine-free diet effectively retards the growth of prostate and breast cancer xenografts in the mouse models. Previous studies of arginine and precursor (glutamate, proline and aspartate)-free diet for 4 weeks showed no side effect in healthy adults, which sheds a light on arginine-restriction diet for cancer therapy. While it may be impractical to follow a strict arginine-free diet, arginine-light diet (such as vegetables, fruits and milk products) could be an alternative choice. It is hoped that an arginine-restricted diet could significantly slow down the growth of ASS1-low tumors and offers benefit in lowering the toxicity of combined chemo- or radio-therapies. Finally yet importantly, as we discussed in the beginning of this review, different tumors utilize different strategies to cope with the nutrient demand or stress, exploring the metabolic heterogeneity of various cancer types definitely is required for personalized medicine [171].

## Figures and Tables

**Table 1:** Summary of arginine transporters (solute carriers) in cancer vs. immune cells.

Cancer		Immune Cells	
SLC	Type	SLC	Type
SLC7A1	Hepatocellular carcinoma [33], colorectal cancer [34], breast cancer [35], leukemia [36]	SLC7A1	memory CD4(+) T cells and CD8(+) T cells [37]
SLC6A14	Colorectal cancer [38], cervical cancer [39], pancreatic ductal adenocarcinomas [40], breast cancer [41]	SLC7A2	Macrophage [42]
SLC7A3	Osteosarcoma [43]	-	-
SLC7A9/SCL3A1	Breast cancer [44]	-	-
SLC214C1	Endometrial cancer [45]	-	-
SLC25A2	Colorectal cancer [46]	-	-

**Table 2:** Comparison of arginine-depleting enzymes.

Comparison of the Enzymatic Properties	Pegylated Arginine Deiminase (ADI-PEG20)	Pegylated Recombinant Human Arginase I (PEG-BCT-100)
Reaction products	L-citrulline + ammonia	L-ornithine + urea
Arginine affinity	High (Km~0.1–1 mM)	Low (Km~2.9mM) [125]
Half-life	7 days [126]	2~3 days [125]
Time requires to maximal arginine depletion in plasma	4 days [127]	4 h [125]
Origin of enzyme [4]	Mycoplasma	Human
Immunogenicity [128]	Antigenic (requires pegylation)	No
Clinical trials (Clinicaltrials.gov)	25 trials Phase I, II, III	10 trails Phase I, II

**Table 3:** ADI preclinical studies.

ADI as a Single Agent				
Type of Cancer	Cell Line	Remarks		Ref
Bladder cancer	T24, J82, UM-UC-3, 5637, RT112, and RT4	ADI-PEG20 reduces the colony formation and cell viability by caspase-independent apoptotic cell death in ASS1-deficient cell lines.		[129]
Breast	MDA-MD-231, ZR-75, T47D, MCF-7, SK-BR-3, MCF-10A	ADI-PEG20 induces the autophagy-dependent cell death, leading to mitochondrial dysfunction and growth inhibition.		[15]
Cholangiocarcinoma	HuCCA, RmCCA-1, BJ-1	ADI-PEG20 treatment reduces cholangiocarcinoma cell viability and proliferation.		[130]
Colon carcinoma, Bladder carcinoma	HCT116, UMUC3	ADI-PEG20 reduces hypoxia-induced NO pathway and vascular perfusion.		[131]
Head and neck cancer	FaDu, HONE-1, KB, OEC-M1, UMSCC-1, SCC-4, SCC-15, SCC-25	ADI-PEG20 inhibits the proliferation of head and neck cancer cells.		[132]
Lymphomas	NcNc, Karpas-422, MyLa	ADI-PEG20 induces the caspase-dependent apoptosis in ASS1-methylated lymphoma cell lines.		[111]
Melanoma	A2058, SK-Mel-2, A375	ADI resistant cell lines are preferentially sensitive to glycolytic inhibitors and glutaminase inhibitors		[23]
Melanoma	A375, A2058, SK-MEL-2	ADI-resistance is due to the induction of ASS1 expression via c-Myc/HIF-1α/Sp4 pathway		[133]
Melanoma, Breast cancer	UCSD354L, UACC62, UACC257, MEL1220, A20558, A375, SK-MEL-2, SK-MEL-5, SK-MEL-624, WM35, WM46, WM1799, WM2664, WM3248, SB-2, MDA-MB-231, SKOV3	ADI-resistance is due to the induction of ASS1 expression via Gas6/Axl/Shp2 signal.		[134]
Melanoma, Breast cancer	A2058, A375, BJ-1, WM2664, BT20, BT549, Hs578T, MDA-MB-157, MDA-MB-231, MDA-MB-436, MDA-MB-453, MDA-MB-468, HCC70, HCC38, HCC1806	Knockdown of GLS increase the sensitivity to ADI-PEG20		[135]
Myxofibrosarcoma	OH931, NMFH-1, and NMFH-2	ADI-PEG20 attenuates the cell viability in ASS1-deficient myxofibrosarcoma cells		[124]
Ovarian cancer	OVCAR3, CAOV3, OVCAR4, IGROV1, TOV112D, OVCAR8, OV90, ES2, TOV21G	The ASS1 expression levels in ovarian cancer cell lines are inversely correlated with the susceptibility to ADI-PEG20		[136]
Pancreatic cancer	MiaPaCa-2, AsPc-1, BxPc-3, Capan1, HPAC, SW1990, L3.6pl, Panc-1	ADI-PEG20 enhances the radio-sensitization by triggering the ER stress pathway, resulting in apoptosis in pancreatic tumor cells.		[137]
Pancreatic cancer	BxPC-3, Capan-I, HPAC, HFAF-II, L3.3, MIA-PaCa-2, Panc-1	ADI-PEG20 inhibits the pancreatic cancer cell growth via induction of apoptosis		[112]
Prostate cancer	CWR22	ADI-PEG20 induces the mitochondrial dysfunction, nuclear DNA leakage, and chromatin autophagy		[16]
Renal cell carcinoma	UOK262	ADI-PEG 20 inhibits the cellular proliferation in fumarate hydratase-deficient cells		[138]
Small-cell lung cancer	GLC1, GLC8, NCI-H1092, NCI-H2141, SBC4, NCI-H82, NCI-H524, NCI-H446, NCI-H889, NCI-H69, NCI-H1963, H1048, DMS53	MYC-driven human SCLC is preferentially sensitive to ADI-PEG20 in vivo		[139]
Small-cell lung cancer	SW1222, SK-LC-13, SE1271, NCI-H82, NCI-H146, NCI-H209, SHP-77, NCI-H740, NCI-H889, NCI-H526, NCI-H69	ADI-PEG20 induces apoptosis and autophagy in ASS1-negative SCLC cell lines		[119]
**Combination treatment with ADI-PEG20**				
**Co-targeting reagent(s)**	**Type of cancer**	**Cell line**	**Remarks**	**Ref**
Chloroquine	Glioblastoma	DBTRG, GAMG, SNB19, U87, U118, CCF, LN229, 8MG, T87G, MO59J, MO59K, 42MG	Combination of chloroquine inhibits autophagy and accelerates ADI-PEG20 induced cell death	[118]
Chloroquine	Sarcoma	Osteosarcoma (U-2 OS, MNNG/HOS, MG-63, NOS-1, HuO 9N2), Leiomyosarcoma (SK-LMS-1, SK-UT-1, SK-UT-1B), Synovial sarcoma (SYO-1, Fuji), Chondrosarcoma (HCH-1), Ewing’s sarcoma (LUPI, RD-ES, SK-ES), Alveolar soft part sarcoma (ASPS-1)	The combination of chloroquine with ADI-PEG20 causes synthetic lethality via necroptosis in sarcoma cell lines	[114]
Cisplatin	Bladder cancer	T24, J82, RT4	Ass1 is down-regulated in cisplatin-resistant bladder cancer cells. The combination with ADI-PEG20 increases the susceptibility and induces apoptosis in cisplatin-resistant cancer cells.	[140]
Cisplatin	Hepatocellular carcinoma	Sk-Hep1, Huh7, Tong, HCC36, Hep3B, Malhavu, PLC5, Huh6, HepG2, SNU398 and SNU182	The combination of cisplatin with ADI-PEG20 suppresses ASS1 expression in HCC cell lines	[141]
Cisplatin	Melanomas	A375, Sk-Mel2, A2058, Mel1220	The combination of cisplatin with ADI-PEG20 enhances the cell death via apoptosis in melanoma cells	[142]
Cisplatin	Small-cell lung cancer, Ovarian cancer, Ovarian adenocarcinoma, Glioblastoma, Melanoma	SCLC, S, H465, SR2, A2780, A2008, A172, A2058	The combination of cisplatin with ADI-PEG20 induces synergistical lethality	[143]
Docetaxel	Sarcoma, pancreatic cancer, and melanoma	SK-LMS-1, SK-UT-1, HTB-93, HT-1080, SK-MEL-2, AS-Pc-1, MiaPaCa-2, MNNG, RDES, and RD HPAC, SYO-1 and FUJI, LUPI, RH28	The combination of docetaxel with ADI-PEG20 overcomes the gemcitabine resistance	[144]
5-Flurouracie	Hepatocellular carcinoma (HCC)	BJ1, A2058, Mel1220, SNU398, SNU387, HepG2, Huh-1	The combination of ADI-PEG20 with 5-FU improves the anti-tumor effect in ASS1-negative HCC cells	[145]
Gemcitabine	Pancreatic cancer	MIA-PaCa2, PANC-1, L3.3	The combination of gemcitabine synergistically enhances ADI-PEG20 anti-tumor effect	[146]
Oxaliplatin	Colorectal cancer	HCT116, SW480, RKO, HT29	The combination ADI-PEG20 with Oxaliplatin shows the synergistic growth inhibition in the ASS1-negative cell lines CRCs	[147]
Paclitaxel	Prostate cancer	CSR22Rv1, PC3, LNCaP	The combination of paclitaxel with ADI-PEG20 retards CWR22Rv1 tumor growth in vivo	[75]
Temozolomide	Glioblastoma	LN229 and U87	The combination of ADI-PEG20 with Temozolomide suppresses the tumor growth irrespective of ASS1 status	[148]
TNF-related apoptosis-inducing ligan (TRAL)	Malignant pleural mesothelioma (MPM)	H211, H290, H2052, H2373, GARD REN, BJ-1	The combination of TNF-related apoptosis-inducing ligan (TRAL) enhances ADI-PEG20 mediated apoptosis in MPM cells	[149]
TNF-related apoptosis-inducing ligan (TRAL)	Melanoma	A375, A2058	The combination of TRAIL with ADI-PEG20 accelerates the cell death in melanoma cell lines	[150]
HAT inhibitor(s)	Melanoma	A2058, K-Mel-2, RCC4	The combination of HAT inhibitors enhances ADI-PEG20 cell killing effect.	[151]
HDAC inhibitor(s)	Pancreatic cancer	Panc1, MiaPaca2, Panc02.03, HS766t, HPAF-II, Suit2, Su8686, Panc03.27, Panc10.05	The combination of HDAC inhibitors with ADI-PEG20 induces the degradation of DNA repair enzyme, C-terminal-binding protein interacting protein (CtIP), resulting in DNA damage and apoptosis.	[82]
BET bromodomain-targeting c-Myc inhibitor	Melanoma	A2058	The combination of ADI-PEG20 with JQ1, a BET bromodomain-targeting c-Myc inhibitor, significantly enhances the killing effect in ADI-resistant cells	[152]
Polyamide inhibitor	Mesothelioma	MSTO, Ju77, H28, H226	The combination of polyamide inhibitor with ADI-PEG20 causes synthetically lethal effect in MPM cells	[87]
PHGDH or GLS inhibitor	Leiomyosarcoma, Melanoma	SKLMS1, SKUT1, SKMEL2	The combination of ADI-PEG20 with PHGDH or GLS inhibitor significantly increases cell death	[22]
N-acetylcysteine	Breast	MDA-MD-231	Combination of N-acetylcysteine with ADI-PEG 20 induces the immunogenic cell death.	[106]
PI3K/AKT inhibitor	Melanoma, Breast cancer	A2058, SK-MEL-2, MDA-MB-231, and A375	The combination of PI3K/AKT inhibitor enhances ADI-PEG20–mediated cell apoptosis.	[24]

**Table 4:** Clinical trials for arginine-depletion enzymes and an arginase inhibitor.

Start Date	NCT Number	Type of Cancer	Treatment	Phase	Status	Ref
Trials for pegylayed arginine deimnase						
Jun-2020	NCT04587830	Glioblastoma Multiforme (GBM)	ADI-PEG20|Temozolomide	Phase 1	Recruiting	
Apr-2019	NCT03922880	Uveal Melanoma	ADI-PEG20|Nivolumab|Ipilimumab	Phase 1	Active, not recruiting	
Jun-2018	NCT03498222	Carcinoma, Non-Small-Cell Lung	ADI-PEG20|Atezolizumab|Pemetrexed|Carboplatin	Phase 1	Withdrawn	
May-2018	NCT03449901	Soft Tissue Sarcoma|Osteosarcoma|Ewing’s Sarcoma| Small Cell Lung Cancer	ADI-PEG20|Gemcitabine|Docetaxel	Phase 2	Active, not recruiting	
Aug-2017	NCT02709512	Mesothelioma	ADI-PEG20 |Pemetrexed and Cisplatin	Phase 2|Phase 3	Recruiting	
Jul-2017	NCT03254732	Advanced Solid Cancers	ADI-PEG20|Pembrolizumab	Phase 1	Active, not recruiting	
Jan-2017	NCT02875093	Acute Myeloid Leukemia	ADI-PEG20|Cytarabine	Phase 1	Terminated	[158]
Jan-2015	NCT01910012	Acute Myeloid Leukemia	ADI-PEG20	Phase 2	Completed	[159]
Nov-2014	NCT02101580	Advanced Pancreatic Cancer	ADI-PEG20 Plus Nab-Paclitaxel and Gemcitabine	Phase 1	Completed	[160]
Nov-2014	NCT02101593	Hepatocellular Carcinoma	ADI-PEG20|Sorafenib	Phase 1	Completed	
Nov-2014	NCT02102022	Advanced Gastrointestinal (GI) Malignancies| Hepatocellular Carcinoma|Gastric Cancer|Colorectal Cancer	ADI-PEG20|modified FOLFOX6	Phase 1|Phase 2	Terminated	[161]
Oct-2014	NCT02006030	Unresectable Hepatocellular Carcinoma	ADI-PEG20|Transarterial chemoembolization	Phase 2	Completed	
Apr-2014	NCT02029690	Pleural Mesothelioma Malignant Advanced|Peritoneal Mesothelioma Malignant Advanced|Non-squamous Non-small Cell Lung Carcinoma|Uveal Melanoma|Hepatocellular Carcinoma|Glioma|Sarcomatoid Carcinoma	ADI-PEG20 With Pemetrexed and Cisplatin	Phase 1	Terminated	[162,163]
Apr-2014	NCT01948843	HER2 Negative Metastatic Breast Cancer	ADI-PEG20|Doxorubicin	Phase 1	Completed	
Dec-2013	NCT01910025	Non-Hodgkin’s Lymphoma	ADI-PEG20	Phase 2	Completed	
Sep-2012	NCT01665183	Cutaneous Melanoma, Uveal Melanoma, Ovarian Carcinoma or Other Advanced Solid Tumors	ADI-PEG20|Cisplatin	Phase 1	Completed	[164]
Dec-2011	NCT01528384	Arginosuccinate Synthetase Deficient	ADI-PEG20	Phase 1	Completed	
Sep-2011	NCT01497925	Solid Tumors|Prostate Cancer|Non-Small Cell Lung Cancer	ADI-PEG20|Docetaxel	Phase 1	Completed	[165]
Jul-2011	NCT01287585	Hepatocellular Carcinoma	ADI-PEG20	Phase 3	Completed	[156]
Jan-2011	NCT01266018	Small Cell Lung Cancer	ADI-PEG20	Phase 2	Terminated	
Jan-2011	NCT01279967	Malignant Pleural Mesothelioma	ADI-PEG20	Phase 2	Unknown status	[166]
Jul-2007	NCT00520299	Metastatic Melanoma|Skin Cancer|Neoplasm	ADI-PEG20	Phase 1|Phase 2	Completed	[127]
Jun-2004	NCT00450372	Melanoma (Skin)	ADI-PEG20	Phase 2	Completed	[167]
Sep-2002	NCT00056992	Carcinoma, Hepatocellular	ADI-PEG20	Phase 2	Completed	
Sep-2001	NCT00029900	Melanoma| Neoplasm Metastasis	ADI-PEG20	Phase 1	Completed	
Trials for pegylated recombinant human arginase and arginase-1 peptide vaccine						
Dec-2018	NCT03689192	Non Small Cell Lung Cancer|Urothelial Carcinoma|Malignant Melanoma|Ovarian Cancer|Colorectal Cancer|Breast Cancer|Squamous Cell Carcinoma of the Head and Neck|Metastatic Cancer	Arginase-1 Peptide Vaccine (ARG1-18,19,20)	Phase 1	Recruiting	
Aug-2018	NCT03455140	Cancer|Pediatric Solid Tumor|Pediatric AML|Pediatric ALL	Pegylated Recombinant Human Arginase (BCT-100)	Phase 1|Phase 2	Recruiting	
Sep-2016	NCT02899286	Relapsed or Refractory Acute Myeloid Leukemia	Pegylated Recombinant Human Arginase (BCT-100)	Phase 2	Unknown status	
Aug-2016	NCT02732184	Acute Myeloid Leukemia|Myelodysplastic Syndrome	Co-ArgI-PEG modified human arginase I	Phase 2	Completed	
Nov-2014	NCT02285101	Melanoma|Prostate Adenocarcinoma	Pegylated recombinant human arginase (PEG-BCT-100)	Phase 1	Completed	[168]
Apr-2014	NCT02089763	Hepatocellular Carcinoma	Pegylated recombinant human arginase	Phase 2	Terminated	
Apr-2014	NCT02089633	Hepatocellular Carcinoma	Pegylated recombinant human arginase|Oxaliplain|Capecitabine	Phase 2	Completed	
Apr-2012	NCT01551628	Leukemia|Lymphoma	Recombinant human arginase 1 Peg5000	Phase 1	Terminated	
Mar-2010	NCT01092091	Neoplasm| Hepatocellular Carcinoma	Pegylated Recombinant Human Arginase I (BCT-100-002)	Phase 1|Phase 2	Completed	[125,169]
May-2008	NCT00988195	Neoplasm| Hepatocellular Carcinoma	Pegylated Recombinant Human Arginase I|Doxorubicin	Phase 1	Completed	
Trials for arginase inhibitor (INCB1158)						
Sep-2019	NCT03837509	Relapsed or Refractory Multiple Myeloma	INCB001158|Daratumumab SC	Phase 1|Phase 2	Recruiting	
Jul-2019	NCT03910530	Advanced Solid Tumors|Metastatic Solid Tumors	Retifanlimab|INCB001158| Retifanlimab + INCB001158	Phase 1	Active, not recruiting	
Mar-2018	NCT03361228	Solid Tumors	INCB001158|Epacadostat|Pembrolizumab	Phase 1|Phase 2	Terminated	
Nov-2017	NCT03314935	Biliary Tract Cancer|Colorectal Cancer|Endometrial Cancer|Gastroesophageal Cancer|Ovarian Cancer|Solid Tumors	INCB001158|Oxaliplatin|Leucovorin|5-Fluorouracil|Gemcitabine|Cisplatin|Paclitaxel	Phase 1|Phase 2	Active, not recruiting	
Sep-2016	NCT02903914	Metastatic Cancer|Solid Tumors|Colorectal Cancer|Gastric Cancer|Head and Neck Cancer|Lung Cancer|Renal Cell Carcinoma|Bladder Cancer|Urothelial Cancer|Mesothelioma	INCB001158|Pembrolizumab	Phase 1|Phase 2	Active, not recruiting	

## Data Availability

No new data were created or analyzed in this study.
